# Role of *Festuca rubra* and *Festuca arundinacea* in determinig the functional and genetic diversity of microorganisms and of the enzymatic activity in the soil polluted with diesel oil

**DOI:** 10.1007/s11356-019-05888-3

**Published:** 2019-07-23

**Authors:** Agata Borowik, Jadwiga Wyszkowska, Anna Gałązka, Jan Kucharski

**Affiliations:** 1 Department of Microbiology, University of Warmia and Mazury in Olsztyn, Plac Łódzki 3, 10-727 Olsztyn, Poland; 2 Institute of Soil Science and Plant Cultivation – State Research Institute, ul. Czartoryskich 8, 24-100 Puławy, Poland

**Keywords:** Phytoremediation, Grasses, Degradation hydrocarbons, EcoPlates, NGS, Bacteria

## Abstract

The objective of this study was to analyze the effect of two grass species, i.e. red fescue (*Festuca rubra*) and tall fescue (*F. arundinacea*), on the functional and genetic diversity of soil-dwelling microorganisms and on the enzymatic activity of soil not polluted and polluted with diesel oil. Grasses were examined for their effectiveness in accelerating degradation of PAHs introduced into soil with diesel oil. A growing experiment was conducted in Kick-Brauckman pots. The soil not polluted and polluted with diesel oil (7 cm^3^ kg^-1^ d.m.) was determined for the count of bacteria, colony development index, ecophysiological diversity index, functional diversity (using Biolog system), genetic diversity of bacteria (using NGS), enzymatic activity, and content of hydrocarbons. Study results demonstrated disturbed homeostasis of soil. The toxic effect of diesel oil on grasses alleviate with time since soil pollution. The yield of the first swath of red fescue decreased by 98% and that of tall fescue by 92%, whereas the yields of the second swath decreased by 82% and 89%, and these of the third swath by 50% and 47%, respectively. Diesel oil diminished also the functional and genetic diversity of bacteria. The use of grasses significantly decreased contents of C_6_-C_12_ (gasoline total), C_12_-C_35_ mineral oils, BTEX (volatile aromatic hydrocarbons), and PAHs in the soil, as well as enabled restoring the microbiological equilibrium in the soil, and increased functional and genetic diversity of bacteria. For this reason, both analyzed grass species, i.e. *Festuca rubra* and *F. arundinacea*, may be recommended for the remediation of soil polluted with diesel oil.

## Introduction

Global urbanization and industrialization have contributed to the severe pollution of the soil environment (Abdel-Shafy and Mansour 2016; Bandowe and Meusel 2017). Among other things, polycyclic aromatic hydrocarbons (PAHs) of petroleum-based products which are accumulated in the soil exert toxic, mutagenic, and teratogenic effect on all organisms of the trophic chain (Huang et al. 2016; Bandowe and Meusel 2017).

Hence, the petroleum-based products have to be eliminated from the natural environment. This may be achieved via mechanical removal, in situ schorching, and the use of chemical agents. Many remediation technologies have been tested to date, with bioremediation acknowledged as safe and cost-effective among them (Allison and Mandler 2018). Truu et al. (2015) and Thijs et al. (2017) recommend phytoremediation as a sustainable approach to soil reclamation. Its use is justified by the fact that plants are autotrophs and as such may develop in polluted areas and at the same time prevent the spreading of pollutants (Cherian and Oliveira 2005). According to Saleem (2016), phytoremediation represents one of the most environment-friendly methods for the biological remediation of soil. It leaves soil structure intact and reduces pollutants migration. Plants, especially the fast-growing ones, contribute to the improvement of the redox potential of the soil, while their root secretions facilitate rhizosphere colonization by microorganisms (Chen et al. 2016). In addition, they aid plant growth and simultaneously lead to degradation and detoxification of pollutants. By this means, the phenomena of rhyzofiltration and rhyzodegradation are likely to occur already in the rhizosphere (Liu et al. 2014; Moubasher et al. 2015). Phytoremediation is another bioremediation technique; however plant used in this process ought to be capable of accumulating contaminants and producing high amounts of biomass. During the phytoremediation process, their root secretions should be highly resistant to unfavorable environmental conditions (Liu et al. 2014). The vast number of roots in the rhizospheric soil constitute sources of organic compounds having various structures that may be used by plants for degradation of such hydrophobic pollutants as TPH, BTEX, and PAHs (Das and Chandran 2011). In addition, decaying root hair provides enzymes to the soil which may also be active in the decomposition of petroleum-based products. The phytoremediation process may, therefore, prove useful in the elimination of petroleum hydrocarbons from the soil environment (Truu et al. 2015).

Literature data (Gałązka and Gałązka 2015; Rashid et al. 2016; Fatima et al. 2018) indicate that petroleum-based products may modify the composition of soil microbiome. Microorganisms most frequently identified in the polluted soil include the following bacteria: *Anabaena fertilissima*, *Bacillus amyloquefaciens*, *Acinetobacter lwofii*, *Bacillus amyloquefaciens*, *Bacillus cereus*, *Bacillus endophyticus*, *Bacillus flexus*, *Bacillus firmus*, *Bacillus licheniformis*, *Bacillus megaterium*, *Bacillus niabensis*, *Bacillus pumilus*, *Bacillus subtilis*, *Comamonas testosteroni*, *Enterobacter cloacae*, *Oceanimonas denitrificans*, *Pseudomonas aeruginosa*, *Pseudomonas brassicacearum*, *Pseudomonas veronii*, *Pseudomonas gessardii*, *Serratia marcescens*, *Shinella granuli*, *Staphylococus sciuri*, *Staphylococus vitulinus*, and *Staphylococcus saprophyticus* (Fatima et al. 2015; Patel et al. 2016; Silva et al. 2015; Wald et al. 2015), as well as mold fungi: *Aspergillus niger*, *Aspergillus oryzae*, *Aspergillus terreus*, *Aspergillus carneus*, and *Penicillium commune* (Díaz-Ramírez et al. 2013; El-Hanafy et al. 2017); and finally yeast: *Candida tropicalis*, *Trichosporon asahii*, *Rhodotorula aurantiaca*, and *Candida ernobii* (Gargouri et al. 2015; Silva et al. 2015). These microorganisms can aid the phytoremediation process. Considering the above, the search for plants effective in the biodegradation of various pollutants, including the petroleum-based products, has become both a scientific and practical challenge. This search may, however, be difficult because the method used for the remediation of habitats polluted with these products should be chosen taken into account oil class, which affects the extent of changes observed in the environment. According to the EPA (2016), diesel oils are divided into four classes: A, B, C, and D. This division is quite arbitrary because when exposed to physical factors in the natural environment, particular classes of diesel oil change their physical and chemical properties, and thus may change their classes.

Degradation of petroleum hydrocarbons in soil depends on nutrient content in the soil and on the natural physicochemical properties of soil (Baptista et al. 2005). Degradation process may be accelerated by using biostimulation (Cosgrove et al. 2005; Taccari et al. 2012), bioaugmentation (Borowik and Wyszkowska 2018b), or phytoremediation (Hou et al. 2015; Thijs et al. 2017). Plants from both the family *Poaceae* and the family *Fabaceae* may prove useful in the phytoremediation of soils polluted with petroleum-based products (Pascale et al. 2016; Cristaldi et al. 2017; Fatima et al. 2018). The main species used to this end include *Avena sativa* (Borowik and Wyszkowska 2018a; Wyszkowska et al. 2015), *Triticum aestivum* (Khan et al. 2018), *Scirpus triqueter* (Zhang et al. 2014), *Scirpus lacustris* (Haritash and Kaushik 2009), *Zea mays* (Borowik and Wyszkowska 2018b), *Medicago sativa* (Agnello et al. 2016), and *Lupinus luteus* (Kucharski and Jastrzębska 2006). Because the effectiveness of phytoremediation is determined by the choice of plant species, we have decided to carry out an experiment assuming that various grass species respond differently to the pollution with diesel oil and that they may accelerate soil environment detoxification by modifying the diversity of soil microbiome. Therefore, the undertaken study aimed to determine the effect of two grass species, i.e., *Festuca rubra* and *Festuca arundinacea*, on the functional and genetic diversity of microorganisms and on the enzymatic activity in soil not polluted and polluted with diesel oil. Red fescue and tall fescue used in the experiment were also analyzed for their effectiveness in accelerating degradation of PAHs derived from diesel oil.

## Material and methods

### Material

#### Soil

Experiment was performed using samples of typical brown soil (Eutric Cambisol) originating from the north-eastern Poland (53.7161 N, 20.4167 E). The main characteristics of soil were presented in Table 1.

**Table 1:** General characteristics of experimental soil

Sand	Silt	Clay	C_org_	N_tot_	pH_KCl_	P	K	Mg	K^+^	Na^+^	Ca^2+^	Mg^2+^	HAC	EBC	CEC	BS
*Ø* μm																
50–2000	2–50	< 2				Available			Exchangeable							
%			mg kg^−1^			mg kg^−1^							mM (+) kg^−1^			%
74.93 ± 5.70	22.85 ± 1.21	2.22 ± 0.26	9.30 ± 0.7	0.62 ± 0.04	6.7 ± 0.5	93.7 ± 8.3	141.1 ± 11.0	42.0 ± 3.9	156.0 ± 13.0	40.0 ± 2.7	623.5 ± 54.0	59.5 ± 4.0	11.4 ± 0.6	49.0 ± 2.9	60.4 ± 3.1	81.1 ± 2.6

*C*_*org*,_ organic carbon content, *N*_*total*,_ total nitrogen content, *HAC*, hydrolytic acidity; *EBC*, sum of alkaline exchangeable cation; *CEC*, exchangeable capacity of the sorption complex; *BS*, saturation with cations

±, standard deviation; *n* = 4.

#### Plants

Two species of plants from the family *Poaceae* differing in their functionality were used for the phytoremediation of soils polluted with diesel oil, i.e.,: red fescue (*Festuca rubra*) of Dark variety and tall fescue (*Festuca arundinacea*) of Rahela variety. The red fescue (Fr) is a small lawn grass with a semi-creeping habit and dark green leaves producing long spikelet flowers, whereas the tall fescue (Fa) is a fodder grass with a high habit and high regrowth dynamics. According to data published, the Research Centre for Cultivar Testing in Poland (COBORU), *Festuca rubra* is capable of biomass production at ca. 4.22 Mg d.m. ha^−1^, whereas *Festuca arundinacea* at ca. 12.97 Mg d.m. ha^−1^.

#### Diesel oil

Diesel oil used in the experiment was BP Diesel with Active technology. It contains low amounts of enriching substances and millions of molecules adhering to contaminants. As assured by the producer, these molecules draw away dirt from elements of the engine, thereby facilitating its work, and adhere to clean surfaces thus forming a protective sheath around the engine (https://www.bp.com/). According to information provided in its safety data sheet, this diesel oil meets requirements set in EC 1907/2006 (REACH).

#### Experimental design

To avoid soil pollution in its natural ecosystem, the experiment was performed in controlled conditions ex situ. Typical brown soil was collected from the depth of 0–20 cm of the topsoil at the Didactic and Experimental Station of the University of Warmia and Mazury in Olsztyn (north-eastern Poland), transported to a greenhouse, mixed, sifted through a screen with mesh size of 1 cm, and then divided into two portions: the first was left unpolluted, whereas the second was polluted with diesel oil with Active technology in a dose of 7 cm^3^ kg^−1^ soil d.m. The experiment was conducted in two series: with unpolluted soil and with soil polluted with diesel oil (DO), in Kick-Brauckman pots (200 mm × 240 mm), in four replications. The pots were filled with 9 kg d.m. of soil not polluted or polluted with DO, depending on the experimental series. Two grass species were used for polluted soil remediation: *Festuca rubra* and *Festuca arundinacea*. The experimental objects were as follows: C—non-sown soil not polluted, CDO—non-sown soil polluted with diesel oil, Fr—soil sown with grasses *Festuca rubra* L., FrDO—soil sown with grasses *Festuca rubra* L. and polluted with diesel oil, Fa—soil sown with grasses *Festuca arundinacea* Schreber, and FaDO—soil sown with grasses *Festuca arundinacea* Schreber L. and polluted with diesel oil. Soil was fertilized with the same mixture containing (in mg kg^−1^ soil d.m.): N—80, P—20, K—40, and Mg—10. The aforementioned elements were used in the following forms: N as CO(NH_2_)_2_, P as KH_2_PO_4_, K as KH_2_PO_4_ and KCl, and Mg as MgSO_4_ · 7H_2_O. Afterwards, the soil was brought to humidity of 50% mwc, and 24 seeds of grasses were sown to each pot. The experiment spanned for 105 days. The grasses were reaped three times: on day 45, 75, and 105 of the experiment. Soil humidity kept at a stable level using distilled water. Throughout the experiment, day time spanned from 13 h and 3 min to 16 h and 31 min, whereas the average air temperature accounted for 15.6 ^°^C and air humidity—for 76.5%. Once the plants had been harvested (III swath), the soil was sifted through a screen with mesh size of 2 mm and subjected to microbiological, biochemical, physicochemical, and chemical analyses.

### Microbiological analyses

#### Bacterial count

Once the growing experiment had been completed, soil samples from each pot were analyzed with the serial dilution method for counts of organotrophic bacteria (Org), ammonifying bacteria (Am), nitrogen-immobilizing bacteria (Im), and actinobacteria (Act) according to the procedure provided in a work by Borowik et al. (2017). The determined bacterial counts were used to compute the colony development index (CD) and the ecophysiological diversity index (EP) according to De Leij et al. (1993).

#### Metagenomics

The metagenomic analysis was carried out based on the hypervariable region V3-V4 of the 16S rRNA gene. Specific sequences of primer 1055F (5′-ATGGCTGTCGTCAGCT-3′) and primer 1392R (5′-ATGGCTGTCGTCAGCT-3′) were used for region amplification and library development. DNA was extracted with a high-performance kit “Genomic Mini AX Soil+” for the genomic DNA isolation from samples. The presence of bacterial DNA in the analyzed soil samples was confirmed using real-time PCR conducted in an Mx3000P thermocycler (Stratagene) with the use of SYBR A Green dye (A&A Biotechnology) as a fluorochrome. The sequencing was performed on an MiSeq sequencer in the paired-end (PE) technology, 2 × 250 bp, using v2 Illumina kit (Genomed S.A. Warszawa, Poland).

#### Functional diversity of bacteria

A Biolog ECO MicroPlate test containing 31 various sources of carbon was used to determine the functional diversity of bacteria in the soil samples. Bacterial activity was analyzed based on the consumption of all carbon sources representing the following groups of compounds: carbohydrates (CB), carboxylic acids and acetic acids (CA&A), amino acids (AC), polymers (PY), and amines/amides (AN). Analyses were conducted as described in a work by Gałązka et al. (2018), using optical density (*λ* = 590 nm) measured in 120 h. The following indices were computed: Average Well-Color Development (AWCD), Shannon-Wiener index (H′), substrate richness (R), and substrate evenness (E).

### Biochemical analyses

Simultaneously with microbiological analyses, the soil samples were subjected to analyses of the activities of seven soil enzymes: dehydrogenases (Deh) with Öhlinger (1996) method, catalase (Cat), urease (Ure), acid phosphatase (Pac), alkaline phosphatase (Pal), β-glucosidase (Glu), and arylsulfatase (Aryl) with Alef and Nannipieri (1998) methods. Activities of all enzymes were determined per 1 kg soil d.m. within 1 h and expressed in the following units: dehydrogenases—μmol TFF (tri-phenylformazan); catalase – mol O_2_; acid phosphatase, alkaline phosphatase, and β– glucosidase – mmol PN (p-nitrophenol); and urease – mmol N-NH_4_. The activity of all enzymes, with the exception of catalase, was determined using a Perkin-Elmer Lambda 25 spectrophotometer (MA, USA). A detailed procedure of enzymatic activity analysis was provided in a work by Borowik et al. (2017).

### Physicochemical and chemical analyses of soil

Both before and after the experiment, soil samples were analyzed for pH in 1 mol KCl dm^−3^, hydrolytic acidity (HAC), sum of exchangeable base cations (EBC), organic carbon content (C_org_), total nitrogen content (N_total_), and contents of available phosphorus, potassium, and magnesium. These analyses were carried out following procedures described in a work by Borowik et al. (2017). In addition, the soil samples were determined for contents of: gasoline (C_6_-C_12_); mineral oils (C_12_-C_35_); volatile aromatic hydrocarbons; benzene; ethylbenzene; toluene; m-, p-, o-xylene; styrene and sum of volatile aromatic hydrocarbons (BETX); naphthalene (NAP); anthracene (ANT); chrysene (CHR); benzo(a)anthracene (BaA); dibenz(ah)anthracene (DahA); benzo(a)pyrene (BaP); benzo(b)fluoranthene (BbF); benzo(k)fluoranthene (BkF); benzo(ghi)perylene (BghiP); indo(123-cd)pyrene (IcdP); and ∑10 PAHs. Contents of PAHs were determined at Weeseling laboratory (Kraków, Poland) on a gas chromatograph coupled with an Agilent 7890A-5975C mas spectrometer equipped with an EI/CI ion source, according to standard methods: ISO 18287 (2006); EN ISO 16703 (2011) and EN ISO 22155 (2016).

### Bioinformatic analysis

The bioinformatic analysis ensuring classification to a species level was carried out with the QIIME package based on a reference sequence database Green Genes v13_8. Reference databases were prepared based on the reference sequence database Greengenes v13_5 Illumina modification, after filtering off sequences with the length shorter than 1250 base pair (bp), filtering off incomplete sequences (lack of classification to the level and species) and sequences containing more than 50 degenerated bases. The sequences filtered in terms of quality were grouped into operational taxonomic units (OTU).

### Statistical analysis

Results achieved were subjected to the statistical analysis using Statistica 13.0 package (Dell Inc. 2016). Homogenous groups were computed with the Tukey test at *P* = 0.05. Distances between clusters were estimated in the analysis of variance using the Euclidean distance acc. to the Ward method. Results were additionally subjected to the principal component analysis (PCA). The index of the effect of diesel oil and the index of phytoremediation effect on soil microbiome were calculated based on formulas described in a work by Borowik and Wyszkowska (2018a). The OTU values which had only one readout in the entire data set and these below 2% were removed from the graphical presentation because they might reflect sequencing errors (Schloss et al. 2011).

## Results

### Plant yield

The response of grasses to soil pollution with diesel oil was explicitly negative (Table 2). The yield of tall fescue (pasture grass) grown on the unpolluted soil was higher, on average, by 65% than that of red fescue (mown grass), but both species exhibited a similar response to soil pollution with DO. Under the influence of DO, the yield of the first regrowth of red fescue decreased by 98% and that of tall fescue by 92%, whereas yields of their second regrowth by 82% and 89%, and yield of their third regrowth by 50% and 47%, respectively. This may be due to the better rooting of plants with time and to the partial degradation of toxic compounds contained in DO.

**Table 2:** Yield grasses, g d.m. pot^−1^

Object	I regrowth	II regrowth	III regrowth
Fr	9.41^b^	12.87^b^	16.53^b^
FrDO	0.17^d^	2.35^d^	8.30^d^
Fa	33.30^a^	54.93^a^	21.99^a^
FaDO	2.72^c^	6.14^c^	11.73^c^

*Fr* soil sown with grasses *Festuca rubra* L, *FrDO* soil sown with grasses *Festuca rubra* L and polluted with diesel oil, *Fa* soil sown with grasses *Festuca arundinacea* Schreber, *FaDO* soil sown with grasses *Festuca arundinacea* Schreber L and polluted with diesel oil. ^a–d^The same letters in the columns indicate homogeneous groups (Tukey’s test, *P* < 0.05; *n* = 4)

### Contents of hydrocarbons in soil

The greatest load of toxic compounds in the soil non-sown polluted with DO (Table 3) was provided by: mineral oils (C_12_-C_35_—4600 mg kg^−1^) and gasoline (C_6_-C_12_—1400 mg kg^−1^). Significantly lesser deposition in this soil was observed for BTEX hydrocarbons, i.e.,: m-, p-, o-xylenese (6680 μg kg^−1^); ethylbenzene (1560 μg kg^−1^); toluene (1450 μg kg^−^1 μg kg^-1^), and benzene (40 μg kg^−1^), as well as for (Table 4) naphtalene (1580 μg kg^−1^), anthracene (97 μg kg^−1^), and chrysene (23 μg kg^−1^), whereas the least one for all other hydrocarbons. Their contents were barely at 5 μg to 12 μg kg^−1^. Sowing grasses onto the polluted soil significantly decreased contents of gasoline and mineral oils, and almost completely eliminated BTEX hydrocarbons from the soil (Table 3).

**Table 3:** Content of gasoline total (C_6_-C_12_), mineral oil (C_12_-C_35_) and volatile aromatic hydrocarbons (BTEX) after the experiment, in 1 kg of d.m. soil

Object	Gasoline total (C6-C12)	Mineral oil (C12-C35)	Benzen	Etylobenzen	Toluen	m-, p-, o-ksylen	Styren	Sum BTEX
	mg		μg					
C	0.8^d^	6^d^	10^b^	10^b^	10^b^	30^b^	10^a^	70^b^
CDO	1400^a^	4600^a^	40^a^	1560^a^	1450^a^	6680^a^	10^a^	9740^a^
Fr	0.8^d^	6^d^	10^b^	10^b^	10^b^	30^b^	10^a^	70^b^
FrDO	189^b^	1900^b^	10^b^	10^b^	10^b^	30^b^	10^a^	70^b^
Fa	0.8^d^	6^d^	10^b^	10^b^	10^b^	30^b^	10^a^	70^b^
FaDO	137^c^	1700^c^	10^b^	10^b^	10^b^	30^b^	10^a^	70^b^

*C*, non-sown soil not polluted; *CDO*, soil polluted with diesel oil; *Fr*, soil sown with grasses *Festuca rubra* L; *FrDO*, soil sown with grasses *Festuca rubra* L and polluted with diesel oil; *Fa*, soil sown with grasses *Festuca arundinacea* Schreber; *FaDO*, soil sown with grasses *Festuca arundinacea* Schreber L and polluted with diesel oil.

^a–d^The same letters in the columns indicate homogeneous groups (Tukey’s test, *P* < 0.05; *n* = 4)

**Table 4:** Content of polycyclic aromatic hydrocarbons (PAH) after the experiment, μg kg^−1^ d.m. of soil

Object	NAP	ANT	CHR	BaA	DahA	BaP	BbF	BkF	BghiP	IcdP	Sum PAH
C	5^c^	5^c^	5^d^	5^b^	5^a^	5^b^	5^c^	5^b^	5^a^	5^a^	50^d^
CDO	1580^a^	97^a^	23^a^	8^a^	5^a^	9^a^	12^a^	10^a^	8^a^	8^a^	1760^a^
Fr	5^c^	5^c^	8^c^	5^b^	5^a^	5^b^	8^b^	6^b^	6^a^	6^a^	59^d^
FrDO	27^b^	14^b^	12^b^	5^b^	5^a^	6^b^	9^ab^	8^ab^	8^a^	6^a^	100^b^
Fa	5^c^	5^c^	9^c^	5^b^	5^a^	5^b^	7^b^	5^b^	5^a^	7^a^	58^d^
FaDO	5^c^	5^c^	12^b^	5^b^	5^a^	8^a^	10^a^	10^a^	7^a^	8^a^	75^c^

*NAP* naphthalene, *ANT* anthracene, *CHR* chrysene, *BaA* benzo(a)antracene, *DahA* dibenz(ah)antracene, *BaP* benzo(a)pyrene, *BbF* benzo(b)fluoranthene, *BkF* benzo(k)fluoranthene, *BghiP* benzo(ghi)perylene, *IcdP* Indo(123-cd)pyrene

The rest of abbreviations are explained under the Table 3

^a–d^The same letters in the columns indicate homogeneous groups (Tukey’s test, *P* < 0.05; *n* = 4)

### Activity of soil enzymes

**Fig 1:**
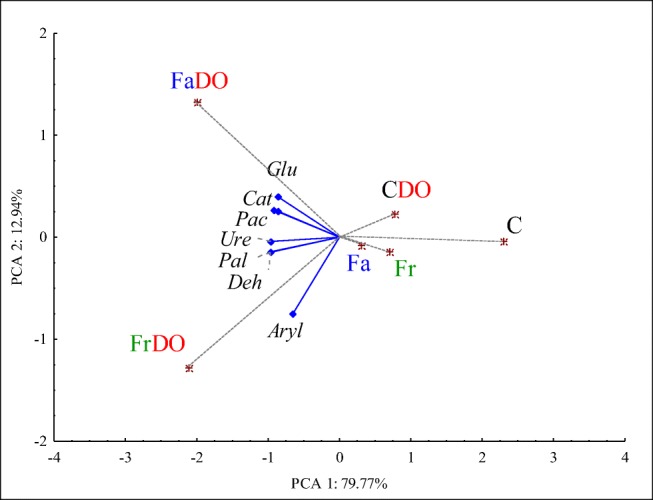
Principal component analysis of activity of soil enzymes. Deh, dehydrogenases; Cat, catalase; Ure, urease; Pac, acid phosphatase; Pal, alkaline phosphatase; Aryl, arylsulfatase; Glu, β-glucosidase; C, soil not polluted; CDO, soil polluted with diesel oil. The rest of abbreviations are explained under the Table 3. Blue diamond, the end of the vector of the primary variable; red asterisk, case

The activity of soil enzymes was another significant element in the assessment of grasses usability in the remediation of soil polluted with diesel oil. Activities of all enzymes tested were determined by PCA1 in 79.77%. Enzymatic activity was negatively correlated with this component in the range from − 0.654 (Aryl) to − 0.974 (Deh, Pal). Distribution of cases indicated the lowest enzymatic activity in the non-sown and non-polluted soil (C) (PCA 1 = 3.452). Its pollution with DO increased its enzymatic activity. The pollution of non-sown soil with DO significantly enhanced activities of dehydrogenases, catalase, urease, β-glucosidase, acid phosphatase, alkaline phosphatase, and arylsulfatase, but the greatest changes were observed in activities of dehydrogenases (an increase by 474%) and urease (an increase by 202%). The enzymatic activity of soil was also increased by soil sowing with grasses. Activities of enzymes were more enhanced by Fa than by Fr (Fig. 1). The highest enzymatic activity was determined in the FrDO pot (PCA1 = − 3.160) and in the FaDO pot (PCA1 = − 2.983).

### Functional diversity of microorganisms

**Fig 2:**
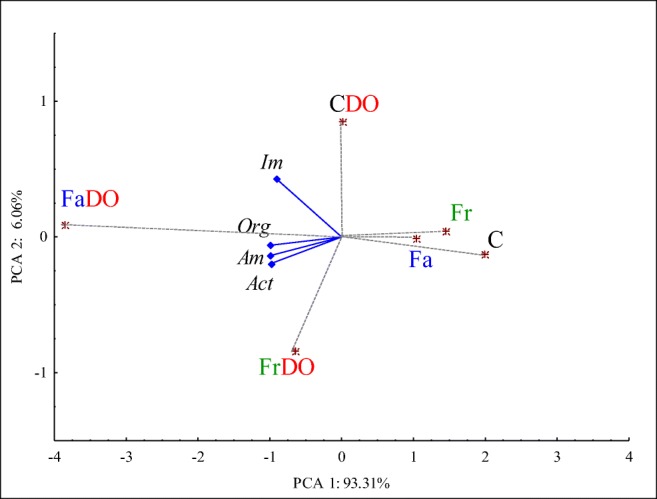
Principal component analysis of number soil microorganisms. Org, organotrophic bacteria; Am, ammonifying bacteria; Im, nitrogen-immobilizing bacteria; Act, actinobacteria. The rest of abbreviations are explained under the Table 3. Blue diamond, the end of the vector of the primary variable; red asterisk, case

The bacterial count (Fig. 2) was negatively correlated with the first principal component in the range from − 0.904 (Im) to − 0.990 (Am and Act). Distribution of cases demonstrates the lowest bacterial count in the non-polluted and non-sown soil (PCA1 = 1.993). The sowing of the non-polluted soil with grasses increased bacterial count in the Fr pot (PCA1 = 1.447) and in the Fa pot (PCA1 = 1.039). Hence, both grass species enhanced bacteria proliferation, however Fa to a greater extent than Fr. A similar observation was made for the activity of soil enzymes.

**Fig 3:**
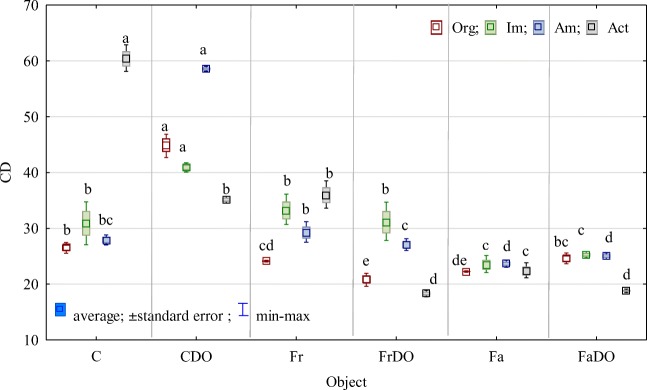
Colony development index (CD) of organotrophic bacteria (Org), ammonifying bacteria (Am), nitrogen-immobilizing bacteria (Im), actinobacteria (Act). The abbreviations are explained under the Table 3 a-e, The same letters indicate homogeneous groups (Tukey’s test, *P* < 0.05; *n* = 4)

Soil pollution with DO not only changed bacterial counts, but in the non-sown soil also altered their structure from slow-growing (k strategists) to fast-growing (r strategists) ones (Fig. 3). An exception were actinobacteria. Values of the CD index achieved in the soil sown with grasses prove that significant changes induced by DO could be alleviated by these plants; however, in the soil sown with red fescue, the CD value significantly decreased upon the effect of DO, whereas in the soil sown with tall fescue, CD value increased compared to the non-polluted soil sown with these grasses. An exception were again actinobacteria, which were predominated by k strategists.

**Fig 4:**
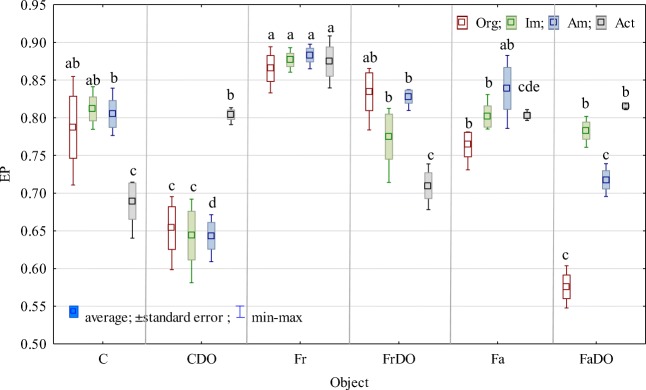
Ecophysiological diversity index (EP) of organotrophic bacteria (Org), ammonifying bacteria (Am), nitrogen-immobilizing bacteria (Im), actinobacteria (Act) The abbreviations are explained under the Table 3 a-e, The same letters indicate homogeneous groups (Tukey’s test, *P* < 0.05; *n* = 4).

Values of the ecophysiological diversity index (EP) prove that DO evoked negative changes in the populations of most of the studied bacteria (Fig. 4). It significantly decreased the diversity of organotrophic bacteria, ammonifying bacteria, and nitrogen-immobilizing bacteria, whereas increased diversity of actinobacteria. These changes were mitigated by both red and tall fescue, except for changes noted in the population of organotrophs in the soil sown with tall fescue.

**Fig 5:**
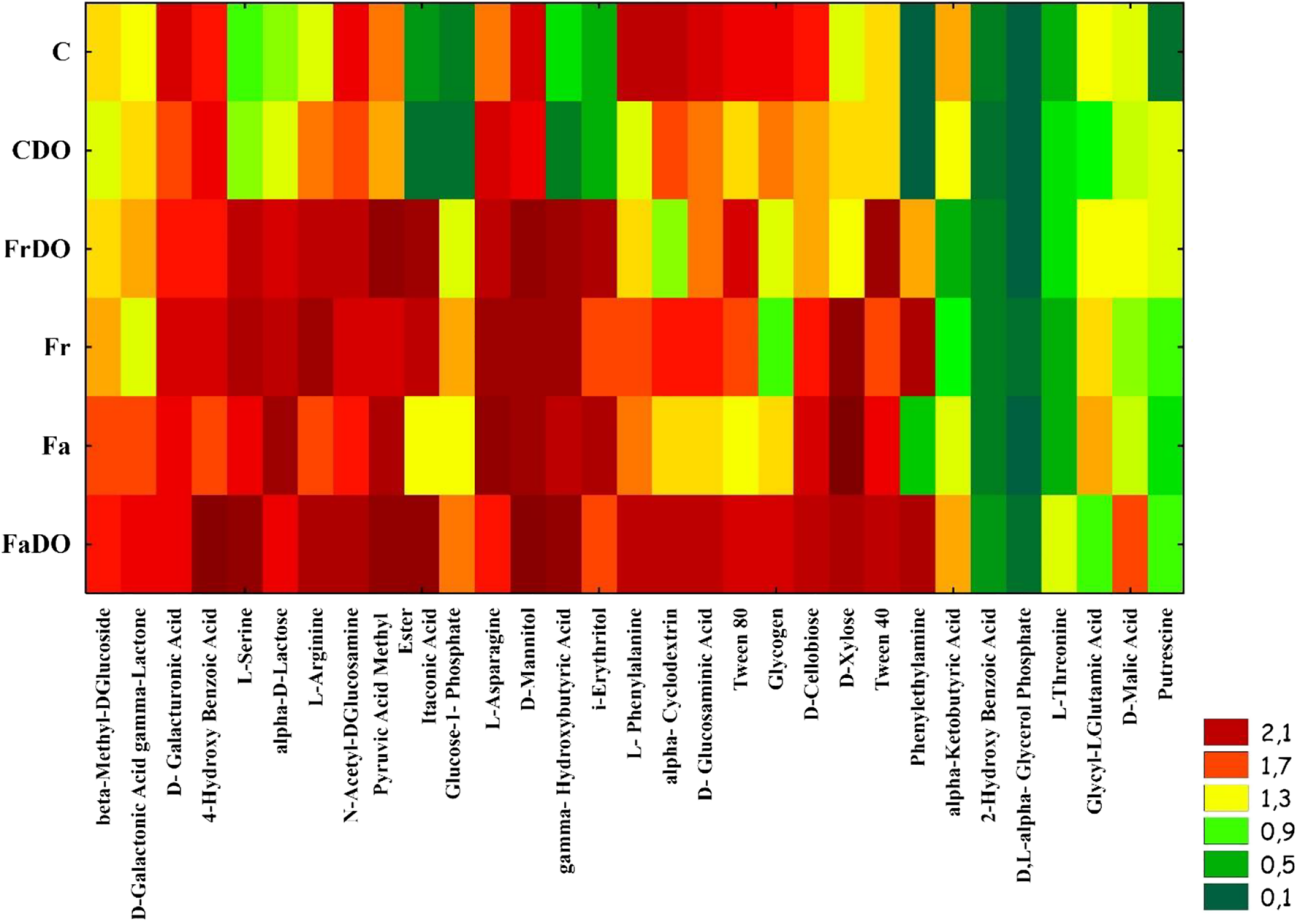
Utilization of carbon sources groups on the microplates EcoPlates®. The abbreviations are explained under the Table 3

**Fig 6:**
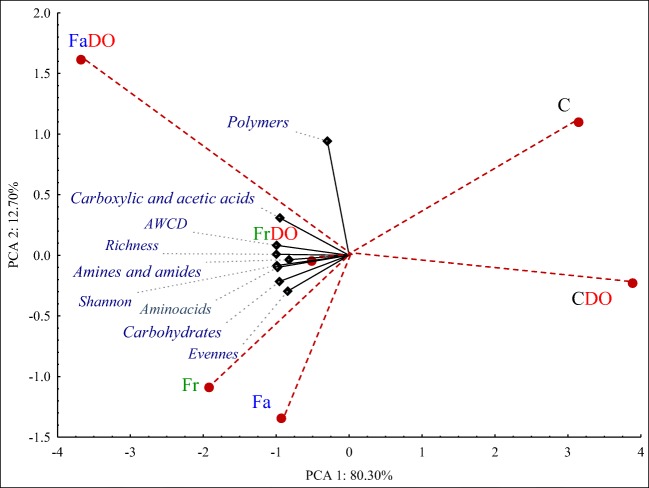
Principal component analysis of utilization of different carbon sources groups on the microplates EcoPlates® The abbreviations are explained under Table 3 Black diamond, the end of the vector of the primary variable;  red circle, case

The functional diversity of bacterial communities may be well described with the Biolog system making use of 31 sources of carbon, in a test conducted on EcoPlate® plates. Of all available carbon sources, bacteria communities were best utilizing: D-mannitol, D-xylose, N-acetyl-D-glucosamine, 4-hydroksy benzoic acid, D-galacturonic acid, D-cellobiose, 4-hydroxybutyric acid, and Tween 40, whereas the slowest consumption by bacteria was observed in the case of DL-a-glycerol phosphate, 2-hydroxy benzoic acid, L-threonine, and putrescine (Fig. 5). Regardless of experimental series, the best carbon sources for bacteria turned out to be carbohydrates and carboxylic acids, whereas significantly worse ones—amino acids and polymers, and the worst ones—amines and amides (Table 5; Fig. 6). The sowing of the unpolluted soil with red fescue and tall fescue contributed to the increased consumption of carbon from amines and amides by 58%, from carbohydrates by 53%, from amino acids by 31%, and from carboxylic acids by 19%. In the case of the polluted soil, respective values accounted for: 98%, 82%, 61%, and 80%. Also values of Well Color Development index (AWCD), Shannon index (H′), and Richness index (R) (Table 6) calculated based on the utilization of chemical compounds by bacteria were the lowest in the non-sown soil, especially in that polluted with DO (Figs. 5 and 6). The PCA analysis showed the strong correlations between the parameters of soil quality and biodiversity indicators. Selected indicators of soil community accounted for 93.0% biological variability in soils (Fig. 6). Red fescue and tall fescue elicited positive effects on bacteria whose functional diversity in the soil sown with grass not only did not diminish under the influence of DO, but was observed to increase.

**Table 5:** Utilization of different carbon sources groups on the microplates EcoPlates®

Object	Amines/amides	Amino acids	Carboxylic acids and acetic acids	Carbohydrate	Polymers
C	0.184 ± 0.018	6.863 ± 0.513	10.752 ± 1.216	11.416 ± 1.053	7.003 ± 0.568
CDO	1.170 ± 0.543	6.420 ± 0.503	9.201 ± 1.775	10.780 ± 0.949	5.886 ± 0.921
Fr	3.046 ± 0.486	9.430 ± 1.030	13.167 ± 1.168	16.930 ± 1.270	5.755 ± 0.407
FrDO	2.556 ± 0.663	8.775 ± 1.113	13.016 ± 0.831	15.975 ± 1.744	6.246 ± 0.853
Fa	1.217 ± 0.072	8.593 ± 0.741	12.376 ± 0.681	17.970 ± 0.735	5.745 ± 0.896
FaDO	3.036 ± 0.986	9.555 ± 0.670	16.280 ± 0.739	18.087 ± 1.484	7.950 ± 0.579

The abbreviations are explained under the Table 3

±, standard deviation; *n* = 3

**Table 6:** Biodiversity indexes. Changes of functional diversities of bacterial communities in soils, as evaluated by the Shannon diversity index (H′), substrate richness (R), substrate evenness (E), and average well-color development (AWCD_590_). Data obtained from the Biolog EcoPlates incubated for 120 h

Object	Shannon (H*′*)	Richness (R)	Evenness (E)	AWCD
C	3.215 ± 0.044	27.00 ± 1.00	0.975 ± 0.007	1.183 ± 0.151
CDO	3.199 ± 0.029	26.66 ± 0.57	0.974 ± 0.003	1.100 ± 0.054
Fr	3.321 ± 0.009	29.33 ± 0.57	0.983 ± 0.006	1.581 ± 0.070
FrDO	3.277 ± 0.033	28.66 ± 1.52	0.977 ± 0.006	1.523 ± 0.172
Fa	3.308 ± 0.016	28.66 ± 0.57	0.986 ± 0.006	1.502 ± 0.042
FaDO	3.346 ± 0.021	30.00 ± 1.00	0.984 ± 0.004	1.794 ± 0.149

The abbreviations are explained under Table 3

±, standard deviation; *n* = 3

### Genetic diversity of microorganisms

**Fig 7:**
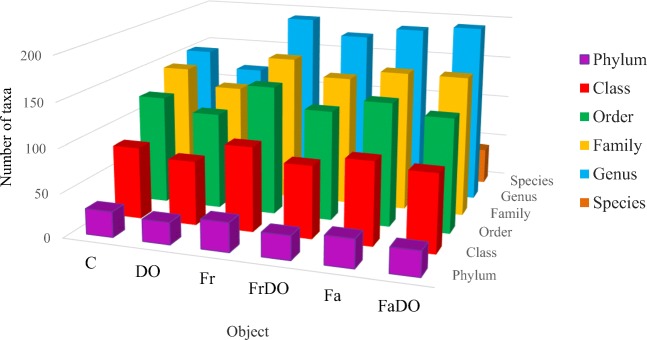
The number of taxa in individual systematic units The abbreviations are explained under Table 3

Soil cultivation type and soil pollution with DO affected its microbiome (Fig. 7). The soil sown with grasses was characterized by a higher number of phyla, classes, orders, families, genera, and species than the non-sown soil. Soil pollution with DO decreased the number of taxa in each taxonomic unit in the non-sown soil and in the soil sown with red fescue. In the case of the soil sown with tall fescue, diminished diversity of bacteria was observed only at the phylum, class, and order level.

Analyses demonstrated that the bacteria belonging to *Proteobacteria* prevailed in both the non-polluted soil (36%) and in the soil polluted with DO (70%), irrespective of grass species. In the non-polluted and non-sown soil, 31% of the bacteria were representatives of *Proteobacteria*, whereas in the soil sown with *F. rubra* and in that sown with *F. arundinacea*, the percentage of *Proteobacteria* bacteria accounted for 38% and 43%, respectively. In the non-sown but DO-polluted soil, these bacteria constituted 75% of the total bacteria count (TBC), whereas in the soil sown with *F. rubra* and with *F. arundinacea*—they represented 68% and 63% of TBC, respectively. Another bacterial phyla having a significant contribution in the microbiome of non-polluted soils were *Actinobacteria* (20%) and *Acidobacteria* (11%), whereas in the polluted soils—*Bacteroidetes* (9%) and *Actinobacteria* (7%).

**Fig 8:**
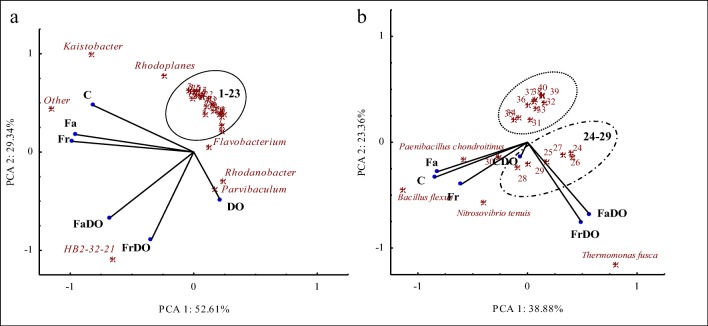
Principal component analysis of genus (**a**) and species (**b**) bacteria with at least 2% of relative abundance in the tested soil samples. 1–23 *Sphingomonas*, *Rhodococcus*, *Pseudomonas*, *Methylibium*, *Alkanindiges*, *Phenylobacterium, Streptomyces*, *Bacillus*, *Candidatus Solibacter*, *Candidatus Koribacter*, *Pseudonocardia*, *Burkholderia, Terracoccus*, *Mycobacterium*, *Nocardioides*, *Devosia*, DA101, *Paenibacillus*, *Geothrix*, *Perlucidibaca*, *Thermomonas*, *Gemmata*, *Ramlibacter*. The abbreviations are explained under Table 3. 24–29 *Pseudomonas nitroreducens*, *Lysobacter brunescens*, *Paracoccus aminovorans*, *Candidatus Koribacter versatilis*, *Sphingomonas wittichii*, *Bosea genosp*. 30–40 *Sphingopyxis alaskensis, Pseudoxanthomonas mexicana*, *Prosthecobacter debontii*, *Sorangium cellulosum*, *Variovorax paradoxus*, *Actinoallomurus iriomotensis*, *Lysinibacillus boronitolerans*, *Bacillus flexus*, *Rhizobium leguminosarum*, *Luteibacter rhizovicinus*, *Rhodococcus fascians.* The abbreviations are explained under Table 3

Bacteria diversity at the genus level was determined by soil pollution with diesel oil (Fig. 8a). In the soil not exposed to DO and sown with both red fescue and tall fescue, the predominating genera included *Kaistobacter*, *Rhodoplanes*, and other. Soil pollution with DO, regardless of its cultivation type, modified its microbiome and contributed to the prevalence of bacteria from *Rhodanobacter* and *Parvibaculum* genera. In addition, sowing these soils with grasses created beneficial conditions for the development of HB2-32-21 bacteria.

The effect of independent variables on the genetic diversity of bacteria is well reflected by the PCA analysis at the species level (Fig. 8b). The *Pseudoxanthomonas mexicana*, *Bacillus firmus*, *Prosthecobacter debontii*, *Sorangium cellulosum*, *Variovorax paradoxus*, *Actinoallomurus iriomotensis*, *Lysinibacillus boronitolerans*, *Rhizobium leguminosarum*, *Luteibacter rhizovicinus*, and *Rhodococcus fascians* bacteria constituted a homogenous group and responded similarly to soil pollution with DO and soil sowing with grasses. In the unpolluted non-sown soil and in the unpolluted soil sown with grasses, the highest OTU number was determined for *Bacillus flexus*, *Paenibacillus chondroitinus*, and *Nitrosovibrio tenuis*. In the DO-polluted soil not sown with grasses, the highest OTU number was reported for *Sphingomonas wittichii* and *Bosea genosp.*, whereas in the soil sown with grasses—for *Thermomonas fusca*, *Pseudomonas nitroreducens*, *Lysobacter brunescens*, *Paracoccus aminovorans*, and *Candidatus Koribacter versatilis*. These species were most probably active in the degradation of toxic compounds of diesel oil, which was indicated by the extent of degradation of C_6_-C_12_ gasoline, C_12_-C_35_ mineral oils, and PAHs (Table 3 and 4). Study results proved that red fescue and tall fescue may be useful in the remediation of soil polluted with diesel oil.

## Discussion

### Plant yield

The exploitation of petroleum-based substances is likely to cause their migration to the soil environment wherein they have a direct influence on plant growth and development (Liu et al. 2014; Zhu et al. 2018; Olaranont et al. 2018). Their presence in the soil environment poses a serious problem because it may lead to changes in the physical and chemical properties of soil (Kucharski and Jastrzębska 2005; Kuppusamy et al. 2017) and to the impaired growth and development of plants (Zhu et al. 2018; Olaranont et al. 2018). Plants that overgrow contaminated areas usually exhibit leaf chlorosis and deformation of the root system (Adam and Duncan 1999). In our study, BP Diesel oil with Active technology had an explicitly negative effect on the growth and development of both grass species studied, i.e., *Festuca rubra* and *Festuca arundinacea*, and caused a significant decrease in their yield. The negative impact of petroleum-based products on plants was also reported by Kaur and Asthir (2015) and Ma et al. (2018). The toxic effect of DO on *Festuca rubra* and *Festuca arundinacea* observed in the present study, as well as on other plant species (Pascale et al. 2016; Olaranont et al. 2018; Fatima et al. 2018; Borowik and Wyszkowska 2018a; Khan et al. 2018) is due to, among other things, disorders in the metabolism of organic compounds in plants (Ma et al. 2018).

### Contents of hydrocarbons in soil

Chemical compounds being constituents of petroleum-based substances are highly capable of accumulating in the soil environment (Truu et al. 2015; Huang et al. 2016; Bandowe and Meusel 2017; Allison and Mandler 2018). They may undergo dispersion or pervade to underground waters (Garcia-Lor et al. 2012). Hence, their effective elimination from the natural environment is necessary. Sowing the contaminated soil with plants having a well-developed root system may prove useful in the elimination of petroleum-based hydrocarbons from the soil environment (Pascale et al. 2016; Olaranont et al. 2018; Fatima et al. 2018; Borowik and Wyszkowska 2018a; Khan et al. 2018). *Festuca rubra* and *Festuca arundinacea* used in the present study turned out effective in degrading contaminants present in diesel oil. In the soil sown with these grass species, gasoline total (C_6_-C_12_) was degraded in 86–90%, mineral oil (C_12_-C_35_)—in 59–63%, sum of BTEX—in 99%, and sum of PAHs—in 94–96%. Also Thijs et al. (2017) suggested phytoremediation to be an effective method for detoxification of soils polluted with petroleum-based products. The positive effect of vegetation on detoxification of soil polluted with hydrocarbons or petroleum-based products was reported by, among others, Soleimani et al. (2010), Song et al. (2015), and Wyszkowska et al. (2015), who ascribed this effect to the mobilization of rhizosphere microbiota (Loss and Yu 2018). Also Abdel-Shafy and Mansour (2016) and Takáčová et al. (2013) demonstrated many bacterial species to be capable of metabolizing various organic compounds. Bacteria and fungi were shown to transform organic compounds to less complex metabolites, and to mineralize them to inorganic compounds, H_2_O, and CO_2_ under aerobic conditions or to CH_4_ in the anaerobic environment (Abdel-Shafy and Mansour 2016). In addition, effective in degrading certain hydrocarbons turned out to be bacteria from the *Terrimonas* and *Burkholderia* genera (Song et al. 2015).

### Activity of soil enzymes

Soil contamination with petroleum-based products upsets its stability. Enzymes that take part in carbon, nitrogen, phosphorus, and sulfur metabolism are very good indicators of soil quality as they reliably depict soil condition in the real time (Adam and Duncan 1999; Wyszkowska et al. 2006; Ma et al. 2018; Nannipieri et al. 2018). Their monitoring provides valuable information about soil health status (Borowik et al. 2017; Wyszkowska et al. 2015). The petroleum-based products usually contribute to an increase in the enzymatic activity of soil (Kucharski and Jastrzębska 2006; Khare and Yadav 2017; Fatima et al. 2015; Wyszkowska et al. 2015); however, this effect depends on product type (Borowik et al. 2017; Kucharski and Jastrzębska 2006). Changes in the activity of individual soil enzymes, determined in the present study, reflected their response to biotic stress induced by soil contamination with BP Diesel oil with Active technology and by cultivation of *Festuca rubra* and *Festuca arundinacea*. This effect of DO on enzymes was also observed in our earlier studies (Borowik and Wyszkowska 2018b; Wyszkowska et al. 2015). Under the influence of red fescue and tall fescue, activities of all soil enzymes increased compared to the control soil (non-sown). Cultivation of these grasses on the soil polluted with DO caused a further significant increase in activities of all analyzed enzymes compared to the non-polluted and non-sown soil. This was due to, among other things, positive effect of grasses and DO on the proliferation of soil microorganisms which are the main sources of soil enzymes (Khare and Yadav 2017; Soleimani et al. 2010; Ma et al. 2018). Part of the enzymes derive from plants, which in our study was documented by the activity of enzymes, affected by red fescue and tall fescue. The higher enzymatic activity in the rhizospheric soil (in soil where *Festuca rubra* and *Festuca arundinacea* were sown) is due to the rhizospheric effect (Wei et al. 2018). This proves that the enzymes, the redox ones in particular, are actively involved in the degradation of organic pollutants (Zhou et al. 2011; Galiulin et al. 2012; Sushkova et al. 2018). These results justify also the usability of the analyzed grass species for the remediation of soil polluted with DO.

### Functional diversity of microorganisms

The petroleum-based products destabilize soil microbiome, causing changes not only in the population numbers of microorganisms but also in their functional diversity (Gałązka et al. 2018; Sun et al. 2012). Literature data (Borowik et al. 2018; Wyszkowska et al. 2015) indicate that, after penetration to the soil, DO usually enhances the proliferation of microorganisms. The inflow of fresh organic matter to the soil usually results in the more intense proliferation of copiotrophic bacteria (Ho et al. 2017), which was probably the case in the non-sown soil polluted with DO. Also in our study were the bacterial counts observed to increase significantly in the soil polluted with DO compared to the non-polluted soil. It is quite obvious, because chemical compounds contained in diesel oil represent important donors of electrons and sources of carbon to multiple bacteria (Wyszkowska et al. 2015). Proliferation of bacteria was also significantly enhanced by the analyzed grasses, with tall fescue having a more enhancing effect compared to the red fescue, which is due the genetic determinants for the production of aerial biomass and thus for the production of underground biomass, which contributes to a stronger rhizospheric effect (Cherian and Oliveira 2005). The highest bacterial count was determined in the case of the coupled effect of grasses and DO, i.e., in the soils sown with grasses and polluted with diesel oil (FrDO and FaDO pots). Differences in the positive effects of both grass species on the bacteria, in favor of tall fescue, were stronger in the soil polluted with DO.

According to Gałązka et al. (2018); Sun et al. (2012); and Borowik et al. (2018), the stability of the soil ecosystem may be established based on the functional diversity of microorganisms using EcoPlates® of the Biolog system. Both, the present study in which soil was exposed to the pressure of BP Diesel oil with Active technology as well as a research conducted by Gałązka et al. (2018), in which soil was polluted with petroleum, demonstrate carbohydrates and carboxylic acids to be the best sources, amino acids and polymers to be significantly worse sources, and finally amines and amides to be the worst sources of carbon to bacteria. Differences in the capabilities of rhizospheric and extra-rhizospheric bacteria for the degradation of chemical compounds are due to the rhizospheric effect (Abdel-Shafy and Mansour 2016).

### Genetic diversity of microorganisms

The petroleum-based products disrupt the microbiological homeostasis of soil, causing changes in its diversity (Hou et al. 2015; Jiao et al. 2016). The analysis of the taxonomic diversity of the Procaryota indicates *Proteobacteria* to be the prevailing taxon in the Phylum rank in soils exposed to the pressure of organic contaminants (Gałązka et al. 2018; Peng et al. 2015; Jung et al. 2016). In the DO-polluted soil analyzed in the present study, *Proteobacteria* accounted for as much as 70% of the total bacterial population. The high percentage of bacteria representing *Proteobacteria*, *Bacteroidetes*, and *Actinobacteria* genera in the microbiome of soil polluted with petroleum-based products is consistent with results of other metagenomic research (Hou et al. 2015).

Considering changes observed in the microbiome of soil polluted with diesel oil, it may be explicitly concluded that in spite of the fact that DO increased the number of OTUs of selected bacterial species, it generally exerted a negative effect on the genetic diversity of soil microbiota. Also Peng et al. (2015) and Hou et al. (2015) claimed that soil pollution with petroleum enhanced the development of certain bacterial communities. In turn, according to Jiao et al. (2016), soil pollution with diesel oil may both enhance and diminish the microbiological diversity. The grass species used in our study, i.e., red fescue and tall fescue, significantly minimized the negative effect of DO on soil microbiome. Changes evoked by DO may result from the fact that its chemical compounds are good nutritive substrates not to all bacteria (Borowik et al. 2017). Another perturbation in this case is the disruption of the physical properties of soil by DO (Varjani 2017).

## Conclusion

Study results demonstrated that soil pollution with diesel oil disturbed its homeostasis. The total yield of the *Festuca rubra* decreased by 72%, whereas the yields of the *Festuca arundinacea* decreased by 81%. Pollution with DO increased enzymatic activity. Activities of enzymes were more enhanced by Fa than by Fr. The higher enzymatic activity in the rhizospheric soil is due to the rhizospheric effect. The positive effect of vegetation on detoxification of soil was ascribed effect to the mobilization of rhizosphere microbiota demonstrated many bacterial species to be capable of metabolizing various organic compounds. The sowing of the unpolluted soil with red fescue and tall fescue contributed to the increased consumption of carbon amines and amides, carbohydrates, amino acids, and carboxylic acids. DO diminished the functional and genetic diversity of bacteria. Microorganisms prevailing in the non-polluted soil included representatives of *Proteobacteria* and *Actinobacteria*, whereas these prevailing in the DO-polluted soil included representatives of: *Proteobacteria* and *Bacteroidetes*.

Sowing the soil polluted with diesel oil with *Festuca rubra* and *Festuca arundinacea* is effective in hydrocarbons degradation and in restoring its microbiological homeostasis.
